# A young man with DiGeorge syndrome and tachycardia

**DOI:** 10.1007/s12471-023-01781-4

**Published:** 2023-05-05

**Authors:** Nicolas Bradt, Leonie Franceus, Alice Fouckova, Becker Alzand

**Affiliations:** 1 Department of Cardiology, AZ Glorieux, Ronse, Belgium; 2 Department of Internal Medicine, UZ Ghent, Ghent, Belgium; 3 Department of Medicine, University of Ghent, Ghent, Belgium; 4 Department of Internal Medicine, AZ Glorieux, Ronse, Belgium

**Fig. 1:**
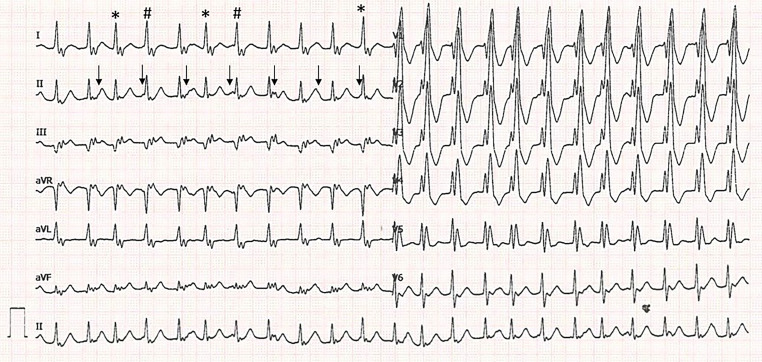
12-lead resting electrocardiogram performed at the emergency department. The *arrows* show the hidden sinus P waves. The *asterisks* show fusion/capture beats. The *hashtags* show the beats that would have reset the tachycardia if there had been retrograde conduction

The 12-lead resting electrocardiogram (ECG) (Fig. 1) shows a broad QRS complex tachycardia, which is due to a supraventricular tachycardia with the pre-existing right bundle branch block. The underlying rhythm is a junctional ectopic tachycardia (JET). Further dissection of the rhythm shows an atrioventricular (AV) dissociation with a ventricular rhythm of 140 bpm and an atrial rhythm of around 95 bpm (Fig. 1, arrows). The slight irregularity in the rhythm is due to occasional capture/fusion beats (Fig. 1, asterisk).

The differentiation of a JET from other supraventricular tachycardias can be challenging, especially from atrioventricular nodal reentrant tachycardia when there is retrograde conduction to the atrium. Our patient has no retrograde conduction and for that reason no resetting of the sinus node with subsequent AV dissociation. This can be seen in Fig. 1 at the fourth and seventh QRS complex (marked with a hashtag). If there had been retrograde conduction these beats would have reset the tachycardia.

**Fig. 2:**
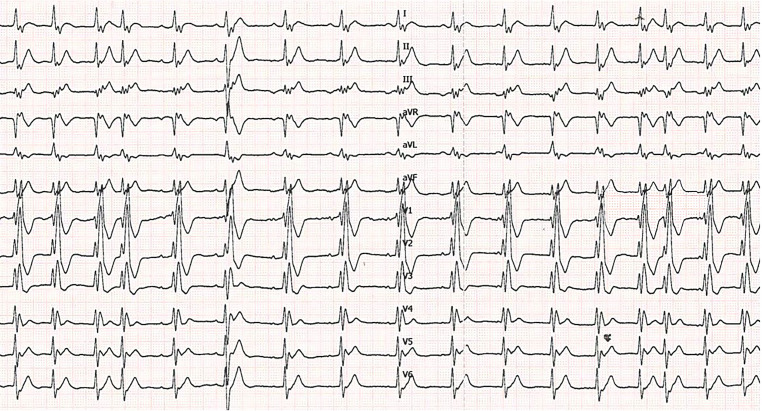
12-lead resting electrocardiogram performed after the administration of 12 mg adenosine

After the administration of adenosine (Fig. 2), the junctional rhythm is blocked while the sinus rhythm resumes but is slowed down. After the short period of normal sinus rhythm, the heart rate gradually increases and the junctional rhythm takes over again. This is referred to as a ‘warm-up pattern’, which typically occurs in a JET in contrast to other supraventricular tachycardias in which the heart rate suddenly increases.

Most cases of JET occur in children [1, 2]. A recent review showed that a JET is the most frequent supraventricular arrhythmia in the perioperative setting [2]. This case shows a JET in an older patient as a possible late complication of extensive cardiac surgery.

An electrophysiological study is needed for the confirmation of the diagnosis. However, our patient declined further examination.
